# The unified protocol for transdiagnostic treatment of emotional disorders for misophonia: a pilot trial exploring acceptability and efficacy

**DOI:** 10.3389/fpsyg.2023.1294571

**Published:** 2024-02-09

**Authors:** Kibby McMahon, Clair Cassiello-Robbins, Anna Greenleaf, Rachel Guetta, Emily Frazer-Abel, Lisalynn Kelley, M. Zachary Rosenthal

**Affiliations:** ^1^Department of Psychiatry and Behavioral Sciences, Duke University Medical Center, Durham, NC, United States; ^2^Triangle Area Psychology Clinic, Durham, NC, United States; ^3^Department of Psychology and Neuroscience, Duke University, Durham, NC, United States

**Keywords:** misophonia, Unified Protocol (UP), single case design, behavioral intervention, cognitive behavioral therapy (CBT), sound intolerance

## Abstract

**Introduction:**

Misophonia is a recently defined disorder characterized by distressing responses to everyday sounds, such as chewing or sniffling. Individuals with misophonia experience significant functional impairment but have limited options for evidenced-based behavioral treatment. To address this gap in the literature, the current pilot trial explored the acceptability and efficacy of a transdiagnostic cognitive-behavioral approach to treating symptoms of misophonia.

**Methods:**

This trial was conducted in two studies: In Study 1, the Unified Protocol for Transdiagnostic Treatment of Emotional Disorders (UP) was delivered to eight patients in order to receive feedback to guide revisions to the treatment to suit this population. In Study 2, ten patients received the revised UP treatment to explore its acceptability and preliminary efficacy. This study used a single-case experimental design with multiple baselines, randomizing patients to either a 2-week baseline or 4-week baseline prior to the 16 weeks of treatment, followed by four weeks of follow-up.

**Results:**

The findings from these studies suggested that patients found both the original and adapted versions of the UP to be acceptable and taught them skills for how to manage their misophonia symptoms. Importantly, the findings also suggested that the UP can help remediate symptoms of misophonia, particularly the emotional and behavioral responses.

**Discussion:**

These findings provide preliminary evidence that this transdiagnostic treatment for emotional disorders can improve symptoms of misophonia in adults.

## Introduction

Misophonia is a recently defined sound intolerance disorder characterized by distressing and functionally impairing responses to certain everyday sounds, commonly those that are oral (e.g., chewing, swallowing, lip smacking) or facial (e.g., nose whistling or sniffling) (Swedo et al., 2022). These sounds and associated stimuli are often called “triggers” of misophonia and are often repetitive sounds produced by other people but can include environmental sounds (e.g., clock ticking, humming of appliances, or silverware on plates) or animal-produced sounds (e.g., animals grooming or eating). In response to hearing or anticipating these sounds, people with misophonia may experience or express a range of aversive responses across physiological, attentional, emotional, cognitive, interpersonal, and behavioral areas of functioning. They often engage in avoidance and escape behaviors to prevent and mitigate exposure to aversive triggering cues. Efforts to avoid misophonia triggers can be very limiting; for example, eating is a daily experience and common source of social interaction. Avoidance of eating due to misophonia sounds can reduce social contact and strain interpersonal relationships. Furthermore, people with functional impairment due to misophonia report higher psychopathology symptoms, lower interpersonal emotion regulation, and lower quality of life than individuals without impairment (Möllmann et al., 2023). Even though misophonia only appeared in the clinical literature as recently as 2001 (Jastreboff and Jastreboff, 2001), research suggests that close to 20% of individuals experience at least moderate symptoms of the condition (Wu et al., 2014; Mattson et al., 2023; Vitoratou et al., 2023). This research suggests that a notable portion of the population may suffer from impairing symptoms of misophonia, highlighting the pressing need for more treatment and prevention efforts. Despite its prevalence and impact, there are few treatment studies examining how to best help those suffering with misophonia. Recent reviews of misophonia identified only three open trials and one randomized controlled trial (Mattson et al., 2023; Rosenthal et al., 2023). The remaining studies were case studies. Given the impact of misophonia, researchers need to continue to identify and rigorously evaluate evidence-based treatments that can help remediate symptoms.

Recent research has highlighted problematic emotional responses as the mechanism underlying impairment in misophonia. People with misophonia report a range of affective responses to their misophonic triggers, especially anger, anxiety and disgust (Dibb and Golding, 2022; Savard et al., 2022; Andermane et al., 2023; Vitoratou et al., 2023). Studies also have pointed to the relationship between misophonia and problems with emotion regulation, particularly difficulties controlling impulsive behavior while upset (Cassiello-Robbins et al., 2020a; Guetta et al., 2022; Rinaldi et al., 2023). Beyond emotional reactions, misophonia is associated with co-occurring psychiatric disorders that are also characterized by the experience of strong emotions such as anxiety and mood disorders (Jager et al., 2020; Rosenthal et al., 2022; Siepsiak et al., 2022). Misophonia does not appear to be reliably linked to any specific disorder, suggesting it is a transdiagnostic problem (Rosenthal et al., 2022). However, the most rigorously conducted study to date exploring co-occurring mental health problems and misophonia using structured diagnostic interviews and larger samples found that, in both children and adults, the most common psychiatric comorbidities are anxiety and mood disorders (Jager et al., 2020; Rosenthal et al., 2022; Guzick et al., 2023). Therefore, one reasonable approach in treatment development for misophonia is to examine interventions that are evidence-based, transdiagnostic (including but not limited to those across anxiety and mood disorders), and target underlying difficulties with emotional functioning across children and adults.

Evidence-based psychological treatments such as cognitive-behavioral therapies (CBTs), are well established treatments, many of which were developed to target changes in emotion regulation. Such CBTs generally help patients change their thoughts and behaviors to manage the onset, experience or reactions to emotions. Preliminary studies using CBTs have begun to show early promise for treating misophonia with CBTs (Rosenthal et al., 2023). An open trial demonstrated that a group delivered CBT led to a significant reduction in misophonia symptoms in 48% of the 90 participants (Schröder et al., 2017). A follow-up study with this treatment using a randomized, waitlist-controlled trial concluded that the brief group-based CBT approach showed both short- and long-term efficacy for misophonia (Jager et al., 2021). Patients in the treatment group reported improvements in mental and physical dysfunction, suggesting the possibility that CBTs might improve areas of functioning in patients with misophonia. A number of case studies also suggest various CBTs could effectively target symptoms of misophonia (Mattson et al., 2023). Findings from these studies indicate promise for the use of cognitive-behavioral approaches to increasing control over attention and affective responses to triggers in treatment of misophonia.

Early studies like these have demonstrated that this disorder can be treated with interventions that help people change their thoughts, physiological responses, behaviors and attentional focus in response to trigger sounds. Because this field of research is in its nascent stage, researchers have ample room to build on these preliminary findings. For example, many of the early uncontrolled case studies and open trials are limited in not using measures of misophonia with strong psychometric properties. Additionally, the CBT protocol tested in the only randomized controlled trial was specifically designed for misophonia symptoms and helping patients change their responses to their trigger sounds. Although this approach is novel and may be quite useful for some people with misophonia, this approach may not target the transdiagnostic processes that may underline misophonia in the context of common co-occurring psychiatric disorders. A CBT-based treatment that improves the ability to respond to emotions skillfully may be helpful in treating misophonia symptoms and, concurrently, other co-occurring mental health problems.

One CBT that can target such emotional processes shared by several diagnostic categories is the Unified Protocol for Transdiagnostic Treatment of Emotional Disorders (UP; Barlow et al., 2018). The UP targets *emotional disorders* a term that transcends diagnostic categories and describes a mechanistic process that contributes to the etiology and maintenance of difficulties managing strong emotions (Bullis et al., 2019). The term “emotional disorders” includes diagnoses such as anxiety, depressive, obsessive-compulsive, trauma-related, and borderline personality disorders. It also includes presenting problems that are not diagnoses in DSM-5 if they can be conceptualized in the described functional model (e.g., dysregulated anger that does not meet criteria for intermittent explosive disorder). Emotional disorders are described by a functional model as: (1) the experience of frequent, intense, predominantly negative emotions; (2) an aversive reaction to the experience of emotions (e.g., the perception that these emotions are intolerable, uncontrollable, unacceptable); and (3) use of avoidance-based emotion regulation strategies (e.g., avoidance, suppression) that typically reduce the intensity of the emotion in the short-term but maintain problems in the long-term (Sauer-Zavala and Barlow, 2014). Over time, this tendency to avoid emotional experiences leads to impairment in daily life (e.g., isolation from others, limiting activities) and even more distress in response to emotions. The conceptualization of emotional disorders provided by the UP maps clearly onto misophonia. Because misophonia can be conceptualized using this functional model of emotional disorders, it is possible the UP serves as a promising candidate treatment for this condition (Rosenthal et al., 2023).

As a treatment, the overarching goal of the UP is to increase acceptance of emotions to reduce reliance on ineffective emotion regulation strategies by teaching CBT skills to manage emotions more effectively. More specifically, this treatment is typically delivered over 16 outpatient sessions and consists of 8 modules. In brief, these modules are (1) motivation enhancement, (2) psychoeducation about the function of emotions and how to track emotional experiences, (3) mindful emotion awareness, (4) cognitive flexibility, (5) changing ineffective behaviors, (6) awareness and tolerance of physical sensations (e.g., interoceptive exposures), (7) emotion exposure, and (8) relapse prevention. These modules are described in detail elsewhere (Payne et al., 2014; Barlow et al., 2018). As described in Table 1, these skills target misophonia in important and distinct ways.

**Table 1:** Core unified protocol modules and their relevance to misophonia.

Module	Module focus	Relevance to misophonia
Mindfulness	Attentional control	Improve ability to deploy attention and reduce hypervigilance toward misophonia cues
Cognitive flexibility	Produce and consider diverse interpretations of situations	Reduce dysfunctional attributions toward self and others
Behavior change	Encourage new ways of responding to strong emotions	Respond to emotions in ways that are consistent with values and goals
Interoceptive exposure	Build awareness and tolerance of physical sensations	Reduce avoidance and escape behavior
Emotion exposure	Reduce avoidance of emotion-provoking stimuli	Engage in valued activities avoided due to misophonia

One previous two-phase study showed promise for using the UP to treat misophonia in children ages 8–16 years old (Lewin et al., 2021). Preliminary results from the first phase with four pilot participants showed modest improvements in evaluator-rated severity of symptoms. The results of this phase concluded that the UP is one possible approach to treatment and suggest that children with misophonia could learn skills to tolerate distress associated with their trigger sounds. The second phase was a randomized clinical trial that compared 10 sessions of the UP to 10 sessions of an emotional/physiological relaxation and education intervention. Outcomes from this study presented at conferences have suggested that compared to those in the comparison group, youth with misophonia who received the UP treatment exhibited greater misophonia symptom improvement (Lewin, 2023). Findings from this pioneering study highlight the potential for using the UP to treat misophonia. Further research in adult populations is the next logical step to studying the UP’s efficacy across all individuals with this disorder.

### Current study

In sum, there is a need for more research on theory-driven, transdiagnostic treatments for misophonia. The UP has been effective with disorders (e.g., anxiety, depressive personality, obsessive-compulsive disorders) that are commonly comorbid with misophonia and share similar mechanisms of emotional dysregulation (Cassiello-Robbins et al., 2020b; Rosenthal et al., 2022). Thus, the UP targets the emotional intolerance, co-occurring disorders, and processes relevant to misophonia. The UP is time-limited and follows a standard protocol, making it accessible and cost-effective to implement with patients. Given its ability to target heterogenous presenting problems, the UP represents an innovative intervention capable of helping patients who experience misophonia as well as co-occurring psychiatric conditions (Rosenthal et al., 2023).

The primary aim of this study is to examine the UP’s applicability to adult patients with misophonia by exploring its acceptability and preliminary efficacy. Two studies evaluated the preliminary promise of the UP for misophonia. In Study 1, we delivered the standard UP to eight patients in order to receive feedback to guide revisions to the treatment for this population. In Study 2, we delivered the revised treatment based on patient feedback from Study 1 and examined the acceptability and preliminary efficacy of the modified UP for misophonia. This study used a single-case experimental design with multiple baselines, randomizing patients to either a 2-week baseline or 4-week baseline prior to the 16 weeks of treatment, followed by 4 weeks of follow-up. This design allowed us to take an in-depth investigation of the between and within-subject effects of the treatment.

## Method

### Participants and recruitment

Patient participants for this study were drawn from a parent study investigating the relationship between misophonia symptoms and medical and psychiatric diagnoses (Rosenthal et al., 2022). Individuals between the ages of 18 and 65 years old enrolled in the parent study by accessing a link on the Duke Center for Misophonia and Emotion Regulation website^1^, which took them to an online screen conducted in REDCap (Harris et al., 2009). The study was approved by the Duke Health Institutional Review Board, and all patients provided signed informed consent to participate. Individuals were recruited from online sources (e.g., searching for information about misophonia, social media, news media stories about misophonia linking to our Center). Individuals who met criteria for a current psychotic disorder, current mania, current anorexia, or were unable to read English were excluded during the online screen. Eligible participants completed the Structured Clinical Interview for DSM-5 (SCID-5; First et al., 2015b) and the Structured Clinical Interview for DSM-5 Personality Disorders (SCID-5-PD; First et al., 2015a) with a trained assessor. In addition, they completed self-report measures including the Misophonia Questionnaire (MQ; Wu et al., 2014). Patients were eligible for the present study if they lived in North Carolina, had a score higher than 2 on the misophonia symptoms scale of the MQ, a score higher than 2 on the misophonia emotions and behavior scale of the MQ, and at least a 7 on the misophonia severity scale of the MQ.

For Study 1, nine people were screened for eligibility for the present study. One person did not qualify based on the MQ score criteria. Therefore, eight patients were enrolled in the first study, which included adults with an average age of 35 years old (*SD* = 13.8), seven women and one man, all white, and mostly single (*n* = 5; 62.5%). For Study 2, 11 people were screened and one person did not qualify based on the MQ score criteria. Thus, 10 patients were enrolled in the second study and included adults who were 33.5 (SD = 11.7) years on average, eight women and two men, six white people, one Native American, two Western Asian, one multi-racial individual, and mostly single (*n* = 6; 60%). Patients from both studies met criteria for a range of psychiatric diagnoses. For more detailed demographic and clinical information on patients in both studies, see Table 2.

**Table 2:** Study 1 and Study 2 patient characteristics.

Study	Patient	Age	Sex	Gender	Race	Ethnicity	Marital Status	Education	Income	Principal Dx	Additional Dx
1	101	33	F	F	C	NH	S	College graduate	0–10 k	MDD	PDD, AG, SOC, GAD, BPD
1	104	54	F	F	C	NH	M	College graduate	>100 k	AG	AFI
1	105	49	F	F	C	NH	M	Master’s degree	65–100 k	None	
1	106	50	F	F	C	NH	P	College graduate	65–100 k	SAD	
1	107	25	M	M	C	NH	S	Some graduate school	20–40 k	None	
1	108	20	F	F	C	NH	S	Some college	10–20 k	SAD	
1	109	23	F	F	C	NH	S	College graduate	20–40 k	OSA	
1	110	26	F	F	C	NH	S	College graduate	40–65 k		
2	201	27	M	M	OA	H	S	Master’s degree	0–10 k	GAD	OSO
2	202	37	F	F	C	NH	D	Some graduate school	40–65 k	GAD	MDD, SOC
2	203	46	F	F	NA	H	M	Some graduate school	>100 k	GAD	PMD, OSO
2	204	45	M	M	C	NH	M	College Graduate	> 100 k	OSA	
2	206	28	F	F	MR	NH	S	College graduate	65–100 k	GAD	AUD, SOC, PTSD
2	207	23	F	F	C	NH	S	College graduate	25–40 k	GAD	CD, PMD, OCD
2	208	27	F	F	C	NH	S	Master’s degree	20–40 k	GAD	
2	209	28	F	F	C	NH	S	Master’s degree	10–20 k	ADHD	MDD, GAD
2	210	19	F	F	OA	NH	S	High school graduate	0–10 k	None	
2	212	55	F	F	C	NH	P	Some college	>100 k	SP	ADHD

Only current and full clinical diagnoses presented. Dx, diagnosis; F, female; M, male; C, Caucasian; MR, more than one other racial group; NA, Native American, American Indian, or Alaska Native; OA, Other Asian or other Asian American (includes India, Malaysia, Pakistan, Philippines); H, Hispanic or Latino; NH, non-Hispanic; D, divorced; M, married, P, living with partner; S, single (never married); Education, highest level of education received; Income, annual household salary in USD; AFI, avoidant/restrictive food intake disorder; AG, agoraphobia; AUD, alcohol use disorder; BPD, borderline personality disorder; CD, cyclothymic disorder; GAD, generalized anxiety disorder; MDD, major depressive disorder; None, no current mental disorder; OSA, other specified anxiety disorder; OSO, other specified obsessive compulsive and related disorder; PDD, persistent depressive disorder; PMD, premenstrual dysphoric disorder; PTSD, posttraumatic stress disorder; SAD, social anxiety disorder; SP, specific phobia.

### Procedure

In Study 1, eight patients received the UP as written in the currently available manual and in Study 2, 10 patients received the UP adapted based on patient and therapist feedback from Study 1. Both studies used a multiple baseline single-case experimental design (SCED; Barlow et al., 2009). This design allows for both within and between subject comparisons. In SCED each patient serves as their own control providing strong internal validity; replication of outcomes across patients provides preliminary external validity.

All patients in both studies completed the initial assessment including the diagnostic assessment (SCID-5 and SCID-5-PD), outcome measures (MQ, OASIS, ODSIS, OAnSIS, DMQ) and other measures beyond the scope of this study via a REDCap online survey. Data from the SCID-5 and SCID-5-PD were entered into REDCap by the assessor and double-checked by a second assessor. Diagnostic data was only used if it was verified, which they were for all patients except one (patient 10 in Study 1) due to missing original paper copies of the SCID-5 interview.

After this initial assessment, patients were randomized to either a two- or four-week baseline phase in which they do not receive treatment but complete the assessment measures at the end of each week via online survey. Randomizing the length of the baseline phase allowed for an observation as to whether change in symptoms occurred when, and only when, the intervention was introduced and controls for changes in symptoms that may occur due to life events or stressors (Barlow et al., 2009).

After the baseline phase, patients completed 16 sessions of the UP (Study 1) or revised UP (Study 2). Treatment in Study 1 was delivered by one of this study’s authors (CCR) who is also an author of the current UP manual and has been certified in the delivery of the UP by Unified Protocol Institute.^2^ Study 2 was delivered by the first author (KM) who was trained in the UP by the first therapist and supervised weekly while delivering the treatment to ensure adherence to the UP. To maintain fidelity to the original protocol of the UP, all patients received 16 sessions of UP. Patients had a 20-week window in which to complete all sessions. Patients completed the outcome measures before each session as an assessment of their symptoms for the week prior.

When all 16 sessions of treatment ended, patients completed exit measures, including the acceptability questionnaire and qualitative interview with a trained assessor (only in Study 1). Then, patients started the four-week follow-up phase. During this phase, patients also complete the online survey with outcome measures at the end of each week. Therefore, patients would have completed the main outcome measures (MQ, OASIS, ODSIS, OAnSIS) once at intake, two or four times in baseline, 16 times during treatment and four times at follow-up for a total of 23 or 25 assessments. Patients completed the DMQ at intake, pre-treatment, week 8, post-treatment, and end of follow-up. The DMQ is a longer measure and was only administered five times to reduce patient burden. At the end of data collection, patients received $10 for each assessment they completed.

### Interventions

The UP (Barlow et al., 2018) is an evidence-based treatment that targets core psychological processes underlying emotional disorders. Specifically, the UP is a 16-session psychotherapy protocol that teaches skills for managing strong emotions to increase acceptance of the emotions. For this study, treatment was delivered over a secure telehealth platform. Importantly, the UP assigns homework between sessions for which patients are expected to practice the skills or read the psychoeducational material in the accompanying workbook. Treatment progress is also assessed and monitored every week with the patients using the weekly outcome measures collected via online self-report questionnaires.

This treatment has eight total modules. Therapists have flexibility regarding how much time they spend in each module; however, for this study they were required to spend at least 4 sessions in module 7 (emotion exposure). The first module, “Setting Goals and Maintaining Motivation,” focuses on patients’ reasons and motivation for change by articulating goals and identifying pros and cons of changing. The second module “Understanding Your Emotions” focuses on teaching patients about the function of emotions in order to understand how emotions are adaptive. In this module patients also learn to break their experience into three components (psychological, cognitive, and behavioral). Finally, patients begin to put their experiences in context by identifying the antecedents to emotions and exploring the short- and long-term consequences of their responses.

The third module, “Mindful Emotional Awareness,” provides psychoeducation about the value of taking a non-judgmental, present-focused awareness of emotions and provides several mindfulness activities that patients practice in order to cultivate this awareness. The fourth module, “Cognitive Flexibility,” teaches patients to identify how they interpret and understand situations and how these interpretations relate to their emotional responses. It then teaches skills for how to generate several alternative interpretations. In the fifth module, “Countering Emotional Behaviors,” patients identify behaviors they employ to avoid or control emotional experiences and which of those are unhelpful for living in line with values. Then they generate different ways of responding behaviorally to intense emotions that are more effective. The sixth module, “Facing Physical Sensations,” focuses on increasing patients’ tolerance of physical sensations associated with intense emotions through the use of interoceptive exposure. In the seventh module, “Emotion Exposure,” patients increase their tolerance of emotional experiences by gradually and repeatedly approaching situations or other triggers that bring up intense emotions. Examples of such situations include watching online videos, having the therapist create the trigger sound in session, or interacting with friends and family members while they create trigger sounds (e.g., at a dinner table). Even if patients still find these triggers aversive, they learn new positive associations with the triggers through inhibitory learning by applying their skills from previous modules. In the eighth and final module, “Moving UP From Here,” patients review the principles of treatment they learned and plan for practicing their skills when they experience intense emotions and symptoms after treatment ends.

In the first study, the UP was delivered according to the existing treatment protocol (Barlow et al., 2018). Upon completion of the UP in Study 1, patients completed a qualitative assessment (see Measures) to provide feedback about their experience in treatment. This information was used to inform edits and adaptations to the treatment delivered in Study 2. Through their qualitative feedback, patients from Study 1 reported not having significant recommendations for improving the treatment beyond the option to receive in-person treatment and the option to extend the 16 sessions to include discussion of other personal issues or longer time on specific skills according to individual needs (see results section). Because both changes would not allow for a controlled investigation of a standard treatment across different patients, the original structure of the UP was maintained. However, the feedback from the study therapist in Study 1 was that her spontaneous generation of misophonia examples in sessions helped patients generalize the UP material to their misophonia symptoms. Therefore, we developed standardized misophonia examples and psychoeducational materials to supplement the existing UP protocol. These materials intended to help patients understand how they can apply the treatment’s descriptions of skills and psychoeducation about emotions like anxiety to their misophonia experiences. A general description of the adaptations to each module and some examples are included in Table 3. Misophonia-specific materials developed for this study are available upon request.

**Table 3:** Adaptations to unified protocol for transdiagnostic treatment of emotional disorders modules and examples in Study 2.

UP module	Adaptations to misophonia	Examples of adaptations
1 Setting goals and maintaining motivation	-Provide an additional description of misophonia and a case example to Chapter 1 of the UP manual. -Encourage patients to generate clear, specific goals for treatment of misophonia symptoms. -Discuss the pros and cons of completing the full 16 sessions of treatment vs. not engaging in treatment	-Case example: A 1-page description of “Eva,” who has suffered from intense anxiety, anger, and avoidance in response to the sound of chewing. -Goals: Eating Sunday dinner with the family; not yelling at spouse when he/she chews gum; going into the office 2x a week where there might be trigger sounds.
2 Understanding your emotions	-Provide psychoeducation of emotions that are common in misophonia. -Provide an additional three-component model to illustrate the emotions, physical sensations, thoughts, and behaviors associated with misophonia. -Provide an additional Antecedents, Responses, and Consequences (ARC) form to illustrate the antecedents and consequences of ways of misophonia responses.	-Emotions: anxiety in anticipation of a trigger sound, anger toward a person generating the sound, guilt and shame in response to unhelpful ways of responding to the sound (e.g., yelling at a person). -Three-component model: anxiety and anger in response to chewing sound during dinner, thoughts like “I cannot stand it when he chews like this,” a physical sensation of clenched jaw, and the behavioral response of leaving the dinner table. -ARC form: highlight short-term distress reduction and long-term loneliness at mealtimes due to misophonia responses.
3 Mindful emotion awareness	-Teach the basic skills as instructed using an additional recorded version of the guided mindfulness exercise from Chapter 7 with the therapist’s voice, inducing emotion with music. -Help the patient plan how to apply mindfulness skills to misophonia experiences.	-Bring present-focused and nonjudgmental attention to trigger sounds (e.g., noticing the timbre and pitch of the sound) or own reactions (e.g., tension in body). -Use anchoring during trigger situations (e.g., meal with friends) to notice reactions before reorienting attention to the conversation.
4 Cognitive flexibility	-Teach cognitive flexibility skills as instructed with the provided ambiguous photo in Chapter 8. -Provide psychoeducation on common thinking traps in misophonia (probability overestimation, catastrophizing). -Explore core beliefs related to misophonia.	-Probability overestimation: assuming a trigger sound will definitely occur and end badly. (e.g., “If I go to the movie theater, someone will definitely eat popcorn behind me and my whole day will be ruined.”) -Catastrophizing: assuming inability to cope healthily with trigger sounds (e.g., “if I hear that sound, I’ll turn into a mess, scream at my spouse and run away like a lunatic.”) -Core beliefs: controlling the environment or inner experiences is always necessary; they are unable to cope with distress or discomfort; they are defective or “broken” because of misophonia and will never improve; and people make trigger sounds to harm them.
5 Countering emotional behaviors	-Provide psychoeducation about how people engage in different emotional behaviors in response to misophonia. -Provide additional examples of emotional behaviors common in misophonia. -Help patients generate alternative behaviors that involve engaging with the sound or misophonia experiences instead of avoiding.	-Avoidance and escape behaviors in misophonia: subtle avoidance: avoiding restaurants, locating sound sources, cell phone use during dinner; cognitive avoidance: ruminating over sound origin; safety signal: carrying headphones everywhere. -Emotional behaviors: leaving the room. -Alternative behaviors: engaging with trigger sounds or experiences.
6 Facing physical sensations	-Provide psychoeducation about the physical sensations common to misophonia. -Practice tolerating those physical sensations by intentionally bringing them up repeatedly.	-Physical sensations: shoulder tension, shallow breath. -Exposure exercise: intentionally squeezing shoulders up to ears for 1 min or breathing into a small coffee straw for 30 s.
7 Emotion exposure	-Provide psychoeducation about exposures for misophonia -Create hierarchy of situations that bring up misophonia experiences and plan skills to use during the exposure.	-The goal of exposures: learning to tolerate misophonia triggers using skills from previous modules (e.g., mindfulness, cognitive flexibility, etc.). -Exposures are introduced gradually with increasingly difficult situations. For example, starting with 30 s of watching an online video of people chewing without sound, then 1 min of watching the same video with sound, then 5 min of observing a close friend while they eat, and finally having an entire meal with the same friend.
8 Moving UP from here	-Identify remaining goals for improving symptoms of misophonia. -Plan to practice skills in service of these goals.	-Remaining goals: address challenging or unavailable situations during treatment, such as big family dinners. -Identifying helpful skills and making concrete plans for practicing them in new situations (e.g., practicing mindfulness skills for 5 min at the same time each morning; setting a phone reminder to practice mindfulness during the big family dinner).

In both studies, the therapists instructed the patients to practice the skills on their misophonia experiences and triggers. Because of the UP’s transdiagnostic approach and the prevalence of comorbid emotional disorders in our sample, patients were also allowed to practice skills on their emotional responses outside of misophonia contexts (e.g., fear of enclosed spaces). If they did so, the therapists made sure that skills were also applied to misophonia or emotions related to misophonia.

## Measures

### Structured clinical interview for DSM-5, research version (SCID-5)

The SCID-5 is a psychometrically validated semi-structured interview used to assess current and lifetime symptoms of DSM-5 disorders (First et al., 2015a,b). Variables used in this study included current and lifetime diagnoses of DSM-5 disorders. All diagnostic variables were coded dichotomously as 0 (did not meet criterion) or 1 (above threshold and met criteria for presence of disorder). Inter-rater reliability was assessed in the parent study (Rosenthal et al., 2022) by a blind rater randomly rating 8% of SCID-I interviews via recorded interviews. Significant Cohen’s κ ranged from 0.63 to 1.00 (all *ps* < 0.05) for most disorders, reflecting acceptable inter-rater reliability. However, due potentially to the low rate of observed values in randomly selected interviews, Cohen’s κ was not significant for lifetime agoraphobia (*κ* = 0.43, *p* = 0.09) or generalized anxiety disorder (*κ* = 0.57, *p* = 0.06).

### Structured clinical interview for DSM-5 personality disorders (SCID-5-PD)

The SCID-5-PD is a semi-structured interview and was used to assess diagnostic symptoms of personality disorders from the DSM-5 by a trained assessor (First et al., 2015a,b). All traits of personality disorders were coded by the assessor as 0 (does not meet criteria), 1 (subthreshold), or 2 (threshold). Severity of symptoms for each disorder was calculated by summing the ratings of 0, 1, and 2 for all diagnostic criteria for each personality disorder. Categorical diagnoses of personality disorders were rated dichotomously as 0 (did not meet criterion) or 1 (met criteria for presence of disorder). Inter-rater reliability was assessed in the parent study (Rosenthal et al., 2022) by a blind rater randomly rating 8% of SCID-5-PD interviews via recorded interviews. Inter-rater reliability on total personality disorder symptoms was evaluated using intraclass correlation coefficients (ICCs) with Cohen’s κ analyses. There was agreement among the different raters for the personality disorders (all *κ* = 1, *p* < 0.001).

### Demographics

A self-report measure developed for this study was used to obtain demographic and descriptive information, including age, ethnicity, marital status, and income.

### Misophonia questionnaire (MQ)

This is a three-part self-report questionnaire that assesses misophonia symptom presence, resulting emotions and behaviors, and the overall severity of sound sensitivities (Wu et al., 2014). The first subscale, the Misophonia Symptom Scale, examines the presence of specific sound sensitivities to different types of sound stimuli (e.g., “people eating,” or “rustling”). Cronbach’s *α* was 0.21 for Study 1 and 0.59 for Study 2 at intake. The second subscale, the Misophonia Emotions and Behaviors Scale, examines emotional and behavioral reactions associated with misophonia. Cronbach’s *α* was 0.71 for Study 1 and 0.43 for Study 2 at intake. The first two parts are rated on a scale from 0 (not at all true) to 4 (always true). The third section, named the Misophonia Severity Scale allows the patient to rate their sound sensitivity on a scale from 1 (minimal) to 15 (very severe).

### Overall anxiety severity and impairment scale (OASIS)

The OASIS contains five items assessing severity and impairment from anxiety; items are scored from 0 to 4 and are summed to provide a total score (Campbell-Sills et al., 2009). Higher scores indicate greater anxiety severity and impairment over the past week. Studies have shown the OASIS to have good internal reliability (Cronbach’s alpha 0.80–0.84; Norman et al., 2006; Campbell-Sills et al., 2009) and one-month test–retest reliability (0.82; Norman et al., 2006). Changes in the OASIS have been found to correlate with changes in other anxiety measures during CBT, suggesting that it is sensitive to change (Moore et al., 2015). Cronbach’s *α* was 0.88 for Study 1 and 0.73 for Study 2 at intake.

### Overall depression severity and impairment scale (ODSIS)

The ODSIS is a modified version of the OASIS designed to capture severity and impairment related to symptoms of depression. Its five items are each scored from 0 to 4 and are summed to calculate the total score (Bentley et al., 2014). Higher scores indicate greater symptoms. A study assessed this measure in three distinct populations and found that the ODSIS has excellent validity with a Cronbach’s alpha of 0.94 in the outpatient sample, 0.91 in the student sample, and 0.92 in the community sample (Bentley et al., 2014). Additional research has suggested that during CBT, the ODSIS is sensitive to change (Osma et al., 2019). Cronbach’s *α* was 0.98 for Study 1 and 0.95 for Study 2 at intake.

### Overall anger severity and impairment scale (OAnSIS; unpublished)

This is a five-item self-report questionnaire based on the OASIS. Items are scored from 0 to 4 with a maximum of 20; higher scores indicate greater anger severity and impairment. To create the OAnSIS, the research team replaced the word “anxiety” with “anger” in the OASIS (Campbell-Sills et al., 2009). No other changes to the measure’s language were made, indicating a high level of similarity between the two questionnaires. As the measure is unpublished, its psychometric properties are currently unknown. Cronbach’s *α* was 0.97 for Study 1 and 0.95 for Study 2 at intake.

### Duke Misophonia Questionnaire (DMQ)

The Duke Misophonia Questionnaire (DMQ) is a psychometrically validated self-report measure of misophonia using factor analytic procedures combined with item response theory in an English-speaking sample (Rosenthal et al., 2021). The DMQ has 86 items and includes subscales: (1) trigger frequency (16 items), (2) affective responses (8 items), (3) physiological responses (5 items), (4) cognitive responses (10 items), (5) coping Before (6 items), (6) Coping During (10 items), (7) Coping After (5 items), (8) Impairment (12 items), and Beliefs (14 items). The composite scale overall Symptom Severity combined Affective, Physiological, and Cognitive Subscales with scores ranging from 0 to 83. Clinical impairment scores (derived from the Impairment Subscale) ranging from 0 to 13 are considered “minimal-mild impairment,” scores between 14 and 38 are considered “moderate impairment,” and scores between 39 and 48 are considered “severe to very severe impairment.” Cronbach’s *α* was 0.93 for the Symptoms subscale, 0.91 for the Impairment subscale, and 0.94 for the Beliefs subscale for Study 1 at intake. For Study 2 at intake Cronbach’s *α* was 0.94 for the Symptoms subscale, 0.70 for the Impairment subscale, and 0.91 for the Beliefs subscale.

### Acceptability measures

Data regarding acceptability of the treatment were collected by qualitative and quantitative self-report measures in both Study 1 and Study 2. All questions were developed by the research team.

In Study 1, the acceptability measure consisted of two quantitative questions answered on a scale of 1 (not at all) to 5 (extremely): (1) How acceptable the treatment was and (2) How satisfied they were with it. Patients also answered qualitative, open-ended questions about their reactions to the treatment, including what they thought of the treatment in general, what were the most helpful parts of treatment, changes they would recommend, and most important things they learned. Responses to these questions were collected by an online survey and interview with a trained assessor.

In Study 2, patients answered three quantitative questions on a scale of 1 (not at all) to 5 (extremely): (1) How acceptable the treatment was; (2) How satisfied they were with it; and (3) How helpful it was for their misophonia. They also answered qualitative, open-ended questions about their reactions to the treatment, including what they thought of the treatment in general, what were the most helpful parts of treatment, changes in misophonia because of the treatment, other changes in their lives or problems because of the treatment, changes they would recommend, and most important things they learned. Responses to these questions were collected by online survey to reduce participant burden that an interview would have added.

### Data analytic plan

To evaluate the acceptability of the UP for patients with misophonia, we derived descriptive statistics for the quantitative questions in the acceptability questionnaires in both studies. We then identified key themes in the patients’ qualitative answers in the online questionnaire and interview with the trained assessor in Study 1. This was done by conducting an informal thematic analysis, guided by Braun and Clarke’s (2006) steps, following the methodology from a study that validated the Amsterdam Misophonia Scale (Naylor et al., 2021). The primary steps included coding the responses to each question and then organizing these codes into broader themes.

To evaluate the impact of the UP on misophonia and symptoms of anxiety, depression and anger, data analyses followed established guidelines for analyzing SCED data using both visual inspection and statistical methods (Barlow et al., 2009). Visual inspection is a conservative approach to analyzing SCED data (Kazdin, 2011). To conduct these analyses, data from the weekly assessments are plotted with lines connecting data points with each phase (baseline, treatment, follow-up), as well as horizontal, dashed lines indicating the mean for each measure within each phase. Changes in the level (i.e., mean) across phases indicate the magnitude of intervention effects. For example, a lower average score on a symptom measure in the treatment phase compared to the baseline phase would indicate a reduction in symptoms. Changes in slope indicate the rate of change. For example, a steeper downward slope in the treatment phase compared to the baseline phase would indicate a faster rate of change in symptoms. Two co-authors (KM and AG) first conducted visual analyses of both Study 1 and Study 2 independently. They then compared their analyses and resolved any discrepancies. The final visual analyses reflect the agreement between both authors.

In addition to the visual inspection, effect sizes were calculated to estimate the magnitude of change on all outcome measures across patients using a *d* statistic developed for SCED studies with correction for small sample sizes (Shadish et al., 2014). These effect sizes were calculated using the DHPS SPSS macro (Shadish, 2015) and with those effect sizes, 95% confidence intervals (CI) were calculated (Shadish et al., 2014). An effect size was considered statistically significant at the alpha level *p* < 0.05 if the CI did not include zero. A *d* of 0.2 was considered a small effect, 0.5 was considered a medium effect, and 0.8 was considered a large effect (Cohen, 1988). Using this strategy, the following comparisons were made: (1) The treatment phase compared to the baseline phase to determine the effects of the UP. (2) The follow-up period compared to the treatment phase to capture any further changes in symptoms after the therapy sessions end. Regarding missing data, one patient did not complete outcome measures once during treatment and another patient did not complete them at three timepoints during follow up in Study 1. In the second study, two patients missed one timepoint during follow up. This missing data was attributed to patients forgetting to fill them out or failing to do so because of life circumstances (e.g., moving houses or job transitions). One patient did not have data for 1 week in baseline due to an administration error. The DHPS SPSS macro treats these missing data with the available data method when calculating effect sizes (Shadish et al., 2014; Peng and Chen, 2021).

Because we administered the DMQ during fewer timepoints than the outcome measures, we only derived descriptive statistics for this measure. We also calculated effect sizes with the Hedges’ correction for the changes from pre-treatment to post-treatment scores without significance testing.

## Results

### Study 1

#### Acceptability

On self-report questionnaires, patients rated the acceptability of the treatment as high (*M* = 4.38, *SD* = 0.74, out of 5). They also rated their satisfaction with the treatment as good (*M* = 3.75, *SD* = 1.04, out of 5). All subsequent responses were gathered from interviews with the patients. In response to the question about what they thought about the treatment overall, patient responses indicated: (1) The treatment was worthwhile completing, helpful, and valuable as it resulted in many positive changes; (2) They learned a lot about misophonia and how it affects their lives; (3) The treatment taught useful skills that had impact on experiences beyond misophonia (e.g., anxiety), and (4) They felt that their skills for managing misophonia had improved as a result of the treatment. Some patients voiced dissatisfaction with elements of the treatment in response to this question, including not liking the basic nature of the skills or feeling frustrated with structured therapy that did not allow for venting.

When asked about what they thought were the most helpful parts of treatment, they said: (1) the individual support from the therapist; (2) homework exercises and worksheets that helped them track their emotions; (3) learning and practicing new skills; and (4) Meeting once a week.

As mentioned previously, the changes they would recommend for the treatment include the option of receiving the treatment in-person or in a hybrid model instead of only telehealth, and that they could have the option to extend the treatment beyond 16 sessions to discuss other personal issues or to further practice skills.

Finally, patients said that the most important things they learned in treatment were: (1) It is possible to get better and reduce symptoms of misophonia; (2) They can use the mindfulness, cognitive, and behavioral skills to manage feelings in response to misophonia; and (3) It’s important to accept emotions. See the Supplementary Table for themes and example quotes.

#### Visual inspection of outcome measures

**Figure 1:**
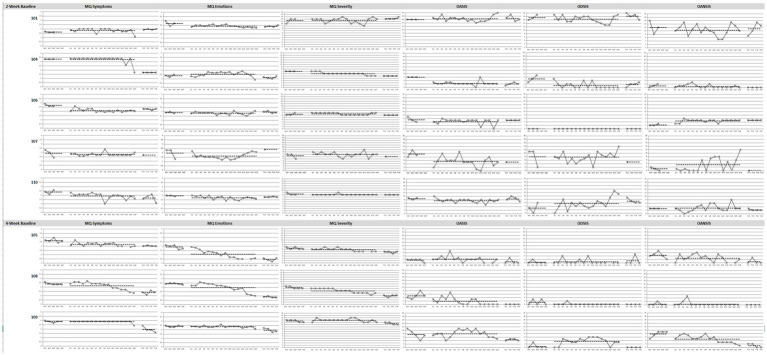
Graphs of Study 1 outcome measures in baseline, treatment and follow-up phases. MQ, Misophonia Questionnaire; OASIS, Overall Anxiety Severity and Impairment Scale; ODSIS, Overall Depression Severity and Impairment Scale; OANSIS, Overall Anger Severity and Impairment Scale. *N* = 8. Patients 101, 104, 106, 107, and 110 were assigned the 2-week baseline and patients 105, 108, 109 were assigned 4-week baseline.

Line graphs for Study 1 outcome measures for each patient are displayed in Figure 1.

##### MQ symptoms

Visual inspection revealed that treatment was associated with reductions from baseline in misophonia symptoms for four patients (105, 106, 108, 110), with a faster rate of change for two patients (108, 110) from baseline to treatment. Patient 108 reported an increasing rate of change toward the last few treatment sessions. During the follow-up phase, two patients continued to maintain their treatment gains (105, 110) and three patients showed improvements from treatment (104, 108, 109). Three patients did not show major changes in misophonia symptoms due to the treatment (104, 107, 109).

##### MQ emotions

Treatment was associated with reductions in emotional and behavioral responses associated with misophonia from baseline for four patients (101, 105, 108, 110). For two of these patients (105, 108), the rate of change was faster toward the end of treatment. During the follow-up phase, two patients continued to maintain their treatment gains (101, 110) and three patients continued to show improvements from treatment (105, 108, 109). One patient showed a worsening of symptoms from treatment (107) but has valid data from only one timepoint at follow up. Of note, patient 107 started experiencing worsening of these symptoms toward the end of treatment. Clinical observation suggested this patient’s worsening was due to the onset of a major depressive episode associated with social stressors. Two patients did not show major changes in emotional and behavioral responses as a result of the treatment (106, 109).

##### MQ severity

Treatment was associated with small reductions in misophonia severity from baseline for two patients (104, 108), with a faster rate of change for one patient, especially toward the end of treatment (104). During the follow up phase, two patients (104, 105) continued to maintain their treatment gains and two patients (108, 109) continued to show improvements from treatment. Five patients did not show major changes in misophonia severity (101, 106, 110, 105, 109).

##### OASIS

Treatment was associated with reductions in anxiety symptoms during the treatment phase for five patients. For three of the patients (104, 107, 108) these changes were large in magnitude and for two patients (106, 110) they were small in magnitude. During the follow-up phase, all five patients maintained their treatment gains (104, 106, 107, 108, 100) and one patient showed improvements compared to the baseline and treatment phases (109). Three patients did not show major changes in anxiety (101, 105, 109), one of which reported low anxiety throughout the study (105).

##### ODSIS

Floor effects were present for patients 105, 106, 108, 109; these patients reported no or minimal symptoms on the ODSIS throughout the study. Treatment was associated with reduction in depression symptoms from baseline for one patient (104) with an increasing rate of change. However, treatment was associated with an increase in symptoms for two patients (110, 109), one of whom experienced these worsening of symptoms toward the end of treatment (110). During the follow up phase, one patient maintained their treatment gains (104), one patient’s symptoms reduced to baseline (109) and one patient’s symptoms got worse from treatment (101). Clinical observation of patients 101 and 110 indicated their symptoms of depression worsened due to interpersonal conflicts and social stressors. Three patients did not show major changes in depression symptoms because of the treatment (106, 107, 105), one of which was due to floor effects as mentioned above (106).

##### OAnSIS

Treatment was associated with reduction in anger from baseline for three patients (101, 105, 109), one of which has a faster rate of change toward the end of treatment (109). On the other hand, there was an increase in symptoms for two patients (106, 107), one of which has an increasing rate of change especially toward the end of treatment (107). During the follow-up phase, one patient continued to maintain their treatment gains (105) and one continued to show improvements from treatment (109). There was a floor effect present for patient 108. Three patients did not show major changes in anger (104, 110, 108).

#### Effect sizes of outcome measures

Effect sizes and 95% confidence intervals for Study 1 are presented in Table 4. UP treatment was associated with significant reductions in emotional and behavioral responses to misophonia that were medium in magnitude. Reduction in misophonia symptoms and anxiety were significant and small in magnitude. Finally, reductions in misophonia severity and anger were significant and small. The treatment was not associated with significant changes in depression. The follow-up phase was associated with medium to large changes in misophonia symptoms, small changes in emotional and behavioral responses to misophonia, anger and misophonia severity, and very small changes in anxiety and depression.

**Table 4:** Mean summary scores and effect sizes for all Study 1 outcomes.

	Baseline	Treatment			Follow-up		
	*M (SD)*	*M (SD)*	*d*	95% CI	*M (SD)*	*d*	95% CI
MQ symptoms	2.51 (0.50)	2.26 (0.58)	0.35	[0.05, 0.65] ^*^	1.84 (0.33)	0.72	[0.63, 1.14] ^*^
MQ emotions	2.11 (0.43)	1.82 (0.50)	0.50	[0.40, 0.92] ^*^	1.54 (0.68)	0.34	[0.25, 0.74] ^*^
MQ severity	8.17 (2.21)	7.85 (2.47)	0.09	[0.07, 0.27] ^*^	7.14 (2.99)	0.27	[0.24, 0.49] ^*^
OASIS	6.93 (4.19)	6.02 (4.93)	0.27	[0.24, 0.52] ^*^	5.34 (5.45)	0.12	[0.10, 0.31] ^*^
ODSIS	3.70 (5.57)	4.46 (5.66)	0.00	[−0.01, 0.17]	4.07 (6.35)	0.04	[0.02, 0.22] ^*^
OANSIS	4.10 (3.94)	3.79 (3.68)	0.05	[0.01, 0.35] ^*^	2.79 (4.03)	0.31	[0.25, 0.64] ^*^

All effect sizes were calculated such that positive *d* values indicate the expected direction of change (i.e., decreases in symptoms). MQ, Misophonia Questionnaire; OASIS, Overall Anxiety Severity and Impairment Scale; ODSIS, Overall Depression Severity and Impairment Scale; OANSIS, Overall Anger Severity and Impairment Scale. *N* = 8.

^*^Significant improvement at *p* < 0.05.

#### Duke Misophonia Questionnaire descriptives

**Figure 2:**
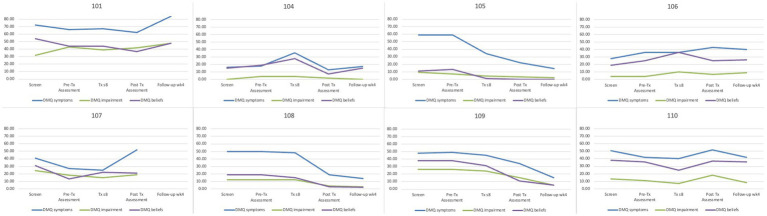
Mean scores from the Duke Misophonia Questionnaire for Study 1 by patient. DMQ Symptoms, Physical Symptom subscale of the Duke Misophonia Questionnaire; DMQ Impairment, Impairment subscale of the Duke Misophonia Questionnaire; DMQ Beliefs, Beliefs subscale of the Duke Misphonia Questionnaire; *N* = 8.

Average scores for the misophonia symptoms, beliefs, and impairment scales from the DMQ are displayed in Table 5. Line graphs are displayed in Figure 2. Patients’ average scores decreased from intake to end of follow up. Specifically, patients reported small changes in symptoms (Hedges’ *g* = 0.28) and impairment (Hedges’ *g* = 0.31) and medium changes in beliefs (Hedges’ *g* = 0.70) in the DMQ from pre to post treatment.

**Table 5:** Mean scores from the Duke Misophonia Questionnaire for Study 1.

Timepoint	DMQ symptoms	DMS impairment	DMS beliefs
Intake	45.63	15.50	28.01
Pre-Tx	43.38	15.63	25.88
Tx Week 8	41.32	14.38	25.25
Post Tx	37.12	13.75	17.63
End of Follow-up	32.29	10.71	18.86

Mean scores from the Symptom Severity (combined Affective, Physiological, and Cognitive subscales), Impairment, and Beliefs subscales from the Duke Misophonia Questionnaire (DMQ; Rosenthal et al., 2021). Timepoints include intake, pre-treatment (right before first session), week 8 of treatment, post-treatment (right after last session) and end of follow-up.

### Study 2

#### Acceptability

On self-report questionnaires, patients rated the acceptability of the treatment (*M* = 4.50, *SD* = 0.53, out of 5), their satisfaction with the treatment (*M* = 4.70, *SD* = 0.48, out of 5), and the helpfulness of the treatment for misophonia (*M* = 4.40, *SD* = 0.52, out of 5) as high.

Patients also wrote about their responses in open text boxes within the online acceptability questionnaire. In response to the question about what they thought about the treatment overall, patients thought: (1) Treatment was worthwhile, helpful and extremely valuable as it resulted in many positive changes, many that were unexpected; (2) The treatment taught very useful information and skills that had impact beyond misophonia (e.g., anxiety); (3) They felt that their skills for managing misophonia had improved as a result of the treatment. However, some patients had some complaints about the treatment, including not knowing how to balance discussing other personal issues and misophonia symptoms during treatment.

Patients reported that the most helpful parts of treatment were: (1) The individual support of the therapist; (2) Homework exercises and worksheets; (3) Learning and practicing new skills; (4) Learning about emotions, how they work and how to identify them; (5) Learning about cognitive flexibility skills; (6) Practicing exposures; and (7) Meeting once a week.

When asked about the specific changes in misophonia symptoms because of the treatment, patients said: (1) Reduced levels of emotions in general and in response to triggers; (2) Reduced emotional and physical misophonia symptoms; and (3) More manageable symptoms as a result of being less reactive to triggers and being able to generate alternative thoughts or actions.

Changes they observed in their social interactions or relationships because of the treatment included: (1) Can spend time with triggering people and settings, including mealtimes; (2) That it was easier to engage in social interactions and relationships; (3) That relationships at home have improved so that they can enjoy their family’s company (including pets and children); and (4) They experienced more positive interactions with others because emotions are less intense.

Furthermore, patients said other changes in their lives or problems because of the treatment included: (1) A better understand what they are feeling and why; (2) The ability to apply skills to manage other symptoms like grief, depression, and anxiety; and (3) Increased hope for the future.

The main change patients recommended for the treatment was the option to add more time to spend on specific skills or other topics, particularly exposures. Otherwise, they reported not having any other major suggestions for ways to change the treatment.

Finally, when asked about the most important things they learned from the treatment, patients said that they learned (1) It is possible to get better and have reduced misophonia symptoms; (2) They can use skills to manage misophonia; (3) The role and function of emotions; and (4) That they are capable of handling more than they think they could. See the Supplementary Table.

#### Visual inspection of outcome measures

**Figure 3:**
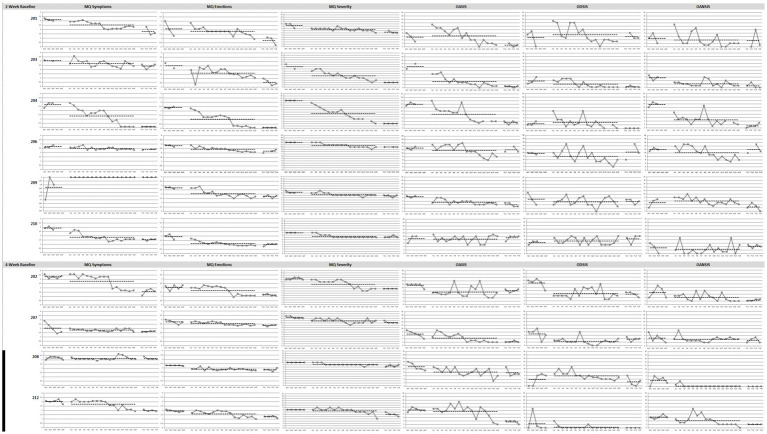
Graphs of Study 2 outcome measures in baseline, treatment and follow-up phases. MQ, Misophonia Questionnaire; OASIS, Overall Anxiety Severity and Impairment Scale; ODSIS, Overall Depression Severity and Impairment Scale; OANSIS, Overall Anger Severity and Impairment Scale. *N* = 10. Patients 201, 203, 204, 206, 209, and 210 were assigned the 2-week baseline and patients 202, 207, 208, and 212 were assigned 4-week baseline.

Line graphs for Study 2 outcome measures for each patient are displayed in Figure 3.

##### MQ symptoms

Visual inspection revealed that treatment was associated with reductions in misophonia symptoms from baseline for seven patients (201, 202, 203, 204, 206, 210, 212), all of whose symptoms changed at a faster rate during treatment than baseline. Two of these patients (202, 212) experienced improvements in the last sessions of treatment. One patient’s symptoms got worse from baseline to treatment (209). From clinical observation, this patient was more socially isolated during the baseline phase and therefore rated sensitivity to certain consonant sounds as lower than during the treatment phase, when she exposed to more people. During the follow-up phase, five patients continued to maintain their treatment gains (203, 204, 206, 210, 212) and two patients showed improvements from treatment (201, 202). The treatment did not change misophonia symptoms for one patient (208).

##### MQ emotions

Treatment was associated with reductions from baseline for 10 patients. For four of these patients (203, 204, 209, 210), these changes were medium in magnitude and for six patients (201, 202, 206, 207, 208, 212) they were small in magnitude. For three of these patients (201, 202, 212), the rate of change was faster toward the end of treatment (201, 202, 212). Furthermore, seven of those patients who improved had a faster rate of change in symptoms in treatment than in baseline (202, 203, 204, 206, 208, 209, 212). During the follow-up phase, seven patients maintained their treatment gains (202, 206, 207, 209, 210, 208, 212) and three patients continued to show improvements from treatment (201, 203, 204).

##### MQ severity

Treatment was associated with reductions from baseline for nine patients (201, 202, 203, 204, 206, 207, 208, 209, 210), eight of which have faster rates of change in symptoms than baseline (202, 203, 204, 206, 207, 208, 209, 210). One patient did not have overall average changes in symptoms from baseline to treatment, but had a reduction in symptoms across the last four sessions (212). During the follow-up phase, 10 patients maintained their treatment gains (201, 202, 203, 204, 206, 207, 208, 209, 210, 212). Treatment was not associated with changes in misophonia severity for one patient (212).

##### OASIS

Treatment was associated with reductions from baseline for seven patients (202, 203, 204, 206, 207, 208, 209) with faster rates of change for six patients (203, 204, 206, 209, 210, 212). Patients 206 and 212 improved particularly in the last sessions of treatment. During the follow-up phase, six patients maintained their treatment gains (201, 203, 204, 207, 209, 212) and six patients had slower rates of improvement from treatment to follow up (201, 203, 204, 206, 207, 212). Three patients did not show major changes in anxiety (201, 210, 212).

##### ODSIS

Floor effects were present for patient 212 as this patient reported no or minimal symptoms on the ODSIS throughout the study except for 1 week during baseline due to personal stressors. Treatment was associated with reduction from baseline for six patients, two of which were larger changes in magnitude (202, 207) and four of which were smaller changes in magnitude (203, 206, 209, 212). Two of those patients had faster rates of change (203, 206) and three others had slower rates of change (202, 207, 209) from baseline to treatment. However, four patients had slight worsening of symptoms from baseline to treatment (201, 204, 210, 208), two of which changed from positive in the baseline phase to negative slope in the treatment phase, or in other words changed from worsening to improving symptoms (204, 208). During the follow up phase, two patients maintained their treatment gains (202, 203) and two patients continued to show improvements from treatment (204, 208). Three patients show a worsening of symptoms (206, 207, 210).

##### OAnSIS

Treatment was associated with reductions from baseline for seven patients (201, 202, 203, 204, 206, 208, 212), all of which had faster rates of change from baseline to treatment. Patient 212 experienced a faster rate of change toward the end of treatment. One patient did not have overall changes in symptoms but the slope changed from positive in baseline to negative during treatment, or from worsening to improving (209). During the follow-up phase, five patients continued to maintain their treatment gains (202, 203, 204, 208, 212). One patient had an improvement in symptoms (209) and another patient showed a worsening of symptoms (206). Although 208 did report changes in symptoms, there was a floor effect as this patient reported no symptoms from third week of treatment until the end of the study. Two patients did not show changes in anger due to the treatment (210, 207).

#### Effect sizes of outcome measures

Effect sizes and 95% confidence intervals for Study 2 are presented in Table 6. In this study, the treatment was associated with large changes in emotional and behavioral responses to misophonia, severity of misophonia, and anxiety, medium changes in anger and misophonia symptoms, and relatively smaller changes in depression. Follow-up was associated with large changes in emotional and behavioral responses to misophonia, medium changes in misophonia symptoms, misophonia severity and anxiety, and small changes in depression. The change in anger was not significant.

**Table 6:** Mean summary scores and effect sizes for all Study 2 outcomes.

	Baseline	Treatment			Follow-up		
	*M (SD)*	*M (SD)*	*d*	95% CI	*M (SD)*	*d*	95% CI
MQ symptoms	2.97 (0.54)	2.54 (0.85)	0.54	[0.45, 0.98] ^*^	2.06 (1.04)	0.50	[0.42, 0.91] ^*^
MQ emotions	2.33 (0.42)	1.79 (0.60)	1.03	[0.89, 1.54] ^*^	1.29 (0.72)	0.81	[0.69, 1.29] ^*^
MQ severity	10.24 (1.69)	8.10 (2.18)	0.96	[0.82, 1.47] ^*^	6.53 (2.76)	0.63	[0.53, 1.07] ^*^
OASIS	10.11 (2.89)	7.30 (3.72)	0.88	[0.75, 1.38] ^*^	5.16 (3.73)	0.59	[0.48, 1.05] ^*^
ODSIS	6.22 (4.37)	4.83 (3.66)	0.33	[0.27, 0.70] ^*^	4.13 (3.82)	0.13	[0.07, 0.49] ^*^
OANSIS	6.22 (3.88)	4.21 (3.55)	0.63	[0.56, 0.99] ^*^	3.00 (3.41)	0.27	[−0.06, 0.61]

All effect sizes were calculated such that positive *d* values indicate the expected direction of change (i.e., decreases in symptoms). MQ, Misophonia Questionnaire; OASIS, Overall Anxiety Severity and Impairment Scale; ODSIS, Overall Depression Severity and Impairment Scale; OANSIS, Overall Anger Severity and Impairment Scale. *N* = 10.

^*^Significant improvement at *p* < 0.05.

#### Duke Misophonia Questionnaire descriptives

**Figure 4:**
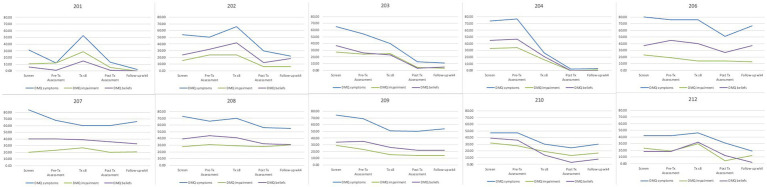
Mean scores from the Duke Misophonia questionnaire for Study 2 by patient. DMQ Symptoms, Physical Symptom subscale of the Duke Misophonia Questionnaire; DMQ Impairment, Impairment subscale of the Duke Misophonia Questionnaire; DMQ Beliefs, Beliefs subscale of the Duke Misphonia Questionnaire; *N* = 10.

Average scores for the misophonia symptoms, beliefs, and impairment scales from the DMQ are displayed in Table 7. Line graphs are displayed in Figure 4. Patients’ average scores decreased from intake to end of follow up. Patients reported large changes in symptoms (Hedges’ *g* = 1.02), impairment (Hedges’ *g* = 1.28) and beliefs (Hedges’ *g* = 1.19) in the DMQ from pre to post treatment.

**Table 7:** Mean scores from the Duke Misophonia Questionnaire for Study 2.

Timepoint	DMQ symptoms	DMS impairment	DMS beliefs
Intake	62.40	24.10	31.90
Pre-Tx	56.10	23.70	32.40
Tx Week 8	51.80	22.80	29.30
Post Tx	33.10	10.80	14.80
End of Follow-up	32.90	11.70	15.60

Mean scores from the Symptom Severity (combined Affective, Physiological, and Cognitive subscales), Impairment, and Beliefs subscales from the Duke Misophonia Questionnaire (DMQ; Rosenthal et al., 2021). Timepoints include intake, pre-treatment (right before first session), week 8 of treatment, post-treatment (right after last session) and end of follow-up.

## Discussion

Misophonia is a disorder characterized by intense aversion to specific repetitive everyday sounds, usually those that are oral or facial and produced by others. This newly defined clinical condition is associated with significant functional impairment, yet there are no established treatments. To address this gap in the literature, this study gathered preliminary support for using the UP (Barlow et al., 2018) to treat misophonia in adults. We conducted two studies. Study 1 delivered the standard version of the UP to eight patients to receive their initial feedback, and Study 2 delivered a version of the UP that has been adapted based on that feedback to 10 patients. Notably, this study used a single-case experimental design to examine the effects of the treatment in depth across a 2 or 4-week baseline phase, the 16-week treatment phase, and a 4-week follow up period. The findings from this study suggested that patients found both the original and adapted versions of the UP to be acceptable and taught them skills for how to manage their misophonia symptoms. Importantly, the findings also suggested that the UP can help remediate symptoms of misophonia, particularly the emotional and behavioral responses. To our knowledge, this is the first study to use this approach to demonstrate the UP’s efficacy for misophonia in adults.

Patients in both study phases indicated that they found the treatment acceptable and helpful in treating misophonia. Because the patients in Study 1 generally responded positively to the standard version of the UP, the general structure of the UP was maintained for Study 2. However, because patients found it useful when the therapist (CCR) specifically applied the transdiagnostic skills and concepts to misophonia symptoms, our team developed standard protocols and materials for misophonia patients. For example, when different emotional disorders are described in chapter one with case description, we provided a standardized description of misophonia and a typical case description of someone who has a strong aversion to the sound of chewing. As a result of these efforts combined, patients learned they can manage their misophonia symptoms with cognitive-behavioral skills. Many patients reported feeling hopeful to experience such an improvement, especially since many of them have suffered from their misophonia all their lives with no evidence-based care.

On the other hand, some patients in both studies expressed a desire for more flexibility in the length of treatment so they could have the option to spend more time on specific modules or skills. From our clinical observations, some patients had difficulty understanding some of the more complex concepts in the UP and other patients had symptoms of other disorders interfere with completing the homework. Although this flexibility was sacrificed to maintain standardization across study participants, future research can explore the UP’s effects on misophonia in real clinical settings where providers can use their clinical judgment to adapt the treatment to patients’ individual needs.

Results from the visual and effect size analyses suggested that the UP was associated with a reduction in multiple misophonia-related outcomes. In Study 1, patients reported moderate improvements in emotional and behavioral responses to misophonia, and small improvements in misophonia symptoms (i.e., sensitivity to different types of sounds and the intensity of the sensitivity) and anxiety as a result of treatment that continued into follow up. Patients in Study 2 reported large changes in emotional and behavioral responses to misophonia, misophonia severity, and anxiety due to the treatment. They also reported moderate improvements in anger and misophonia symptoms. These changes mostly continued into follow up. Therefore, the most robust effect may be the UP’s ability to improve the way patients reacted to misophonia cues behaviorally and emotionally. Because these effects were stronger than those on misophonia symptoms, these findings combined highlight how this treatment can teach patients to react differently and cope effectively with their misophonia even if they still find their trigger sounds uncomfortable. These findings are in line with Lewin et al.’s (2021) study that demonstrated how youth with misophonia benefitted from learning skills to manage their symptoms. Furthermore, the pattern of findings was robust across both studies even though some of the effects in Study 2 were larger than those in the first study. Some of the differences in the findings between studies could be attributable to differences between the therapists. Of note, because the therapist in Study 1 (CCR) supervised the therapist in Study 2 (KM), patients in the second study benefitted from two therapists’ input and the clinical insights from the first study. Although there was not enough power to conduct hypothesis testing with the DMQ, the descriptive data on the DMQ also showed improvements in symptoms, impairment, and associated beliefs in this sample. On the other hand, the treatment did not have such effects on depression symptoms. However, this finding may be due to the low rates of depressive symptoms in this particular sample. Floor effects also explained why some of the patients across both studies did not report improvements due to the treatment. Other patients who did not show notable improvements in misophonia may have had comorbid conditions (e.g., ADHD, trauma) or other life circumstances that interfered with learning and practicing the skills. More research with large, diverse samples is needed to determine if there are systematic reasons why the UP may be contraindicated for some potential patients.

Although the UP teaches similar cognitive- behavioral skills taught in the other previous CBT studies for misophonia (Schröder et al., 2017; Jager et al., 2021; Rosenthal et al., 2023), this treatment follows a curriculum of eight modules that culminates in inhibitory learning-based exposures. The sequence of modules builds on itself, as patients gradually gain new insight into their thoughts, avoidance behaviors, and physical sensations in response to sounds and slowly learn how to engage differently. For example, patients often became aware of beliefs that drove misophonia, such as not being able to tolerate the discomfort, the discomfort will last “forever,” or it would lead them to engage in behaviors that would damage their lives (e.g., “I’ll scream at my parents and ruin my relationship with them”). Patients then challenged these beliefs directly during the later sessions when they practiced skills like mindfulness during exposures, such as while watching an online video of people chewing. In these instances, they learned that they can respond to aversive sounds differently than their habitual freeze, flight, or fight responses.

This approach of building skills prior to engaging in inhibitory learning exposures might explain why several patients experience large improvements in their symptoms within the final sessions of the treatment. Our patients’ generally positive response to exposures were surprising given the negative perceptions of exposures as a treatment option for misophonia (Smith et al., 2022). However, in this study, many of our patients endorsed core beliefs that their discomfort is intolerable and they would be unable to cope with it without avoidance. Thus, exposure in the UP allowed them to challenge this belief by engaging in a situation where they encountered the sound while using their skills and learned whether the distress was truly intolerable or unending. If they had been treated with habituation-based exposure methods instead (i.e., being presented their trigger sounds repeatedly with the expectation that distress will extinguish over time), experiencing their discomfort and their maladaptive coping mechanisms might provide further evidence for these beliefs. In contrast, patients treated with the UP are specifically instructed to practice their skills during exposures, which create new experiences through inhibitory learning (Craske et al., 2014). While the use of habituation-based exposure is not indicated with this population at this point due to patient lack of acceptability (Smith et al., 2022), exposures using an inhibitory learning model for misophonia have been proposed for use by others (Frank and McKay, 2019). Thus, the UP’s approach to exposures might be particularly beneficial for patients with misophonia, similar to interventions in the group-based CBT Schröder et al., 2017; Jager et al., 2021 that involved pairing trigger sounds with new stimuli, positive memories or emotions. From our anecdotal experience and casual feedback from the patients, they were receptive to the exposure module in the UP because the stated purpose was to “practice their skills in situations that feel challenging or bring up intense emotions.” By the time they reached this final module, many patients had already practiced their skills in misophonia contexts on their own or felt enough mastery to engage in exposure by then. Patients who felt less confident benefitted from the freedom to choose their own exposure assignments.

Our findings also revealed some potential drawbacks of the UP for misophonia. Some patients found the structured nature of the treatment challenging and reported having difficulty balancing discussions of their misophonia symptoms versus other symptoms or life challenges in therapy. The UP is designed as a transdiagnostic treatment, so patients were able to apply skills to misophonia or other issues as they learned them. However, many patients struggled with other comorbid conditions such as attention deficit/hyperactivity disorder or trauma symptoms that were either more impairing or interfered with acquisition of the material. More research is needed to understand if targeting other symptoms or disorders with evidence-based treatments before addressing misophonia would be beneficial. In addition, providers can implement the UP in a more flexible way by targeting other symptoms or adjusting the length of each module to suit the individual needs of their patients. Although such flexibility is difficult in controlled research, implementing and studying the UP for misophonia in non-research clinical settings can shed light on how this flexibility would influence outcomes. Alternatively, it may be useful for future research to explore the use of other transdiagnostic evidence-based treatment frameworks capable of treating the co-occurring combination of misophonia and a range of mental health problems. Process based therapy (PBT; Hofmann and Hayes, 2019) is one candidate model to consider, as such an approach uses interventions to target, in a personalized and patient empowered manner, the network of interrelated biological, psychological, and/or social processes underlying a given individual’s impairment (Rosenthal et al., 2023).

Although the findings from this study provide promising support for the use of the UP (especially the adapted version) for treating misophonia, this study had several limitations that are worth noting. First, while the single case experimental design allowed for rigorous and thorough assessment of the treatments’ effects on individual patients, the sample sizes were small. The small sample sizes allow for outliers to bias the results, which may have led to significant differences for most of the comparisons. Replication of these results with larger sample sizes using different controlled trial methodology is the logical next step to this line of research. Second, the treatment was delivered by only two therapists, one in the first study and another in the second study. Both therapists were Ph.D.-level clinical psychologists trained in delivering cognitive behavioral therapies, which may not represent the approach of all the community providers who may treat patients with misophonia. More research with multiple study therapists is needed to understand the effects of the treatment itself across a range of providers. Third, we did not include established measures of therapy satisfaction questionnaires, such as the Client Satisfaction Questionnaire (Attkisson and Zwick, 1982). Finally, a limitation of this study is the low Cronbach’s alphas for the symptoms and emotional and behavioral responses (for Study 2) subscales of the Misophonia Questionnaire at the intake session. These poor scale reliability results may be attributed to the small sample size, but they can also suggest that patients with misophonia may have different profiles. For example, some patients in our study endorsed strong reactions to specific types of sounds (e.g., chewing) but low or no reactions to other types of sounds, which made the interitem correlations among the symptoms subscale of the MQ very low. In addition, there is emerging research suggesting that misophonia can manifest in either internalizing and externalizing presentations (Vitoratou et al., 2023) so patients in our study who reacted with fear, panic and shame did not endorse the items in that subscale that capture aggressive responses. Again, studies with larger samples and other measures of misophonia are needed to determine the UP’s efficacy for different aspects of the disorder.

Third, at the time of study development the MQ was a predominant self-report measure of misophonia (Wu et al., 2014). The MQ, however, is limited by its focus on presence of triggers and frequency of emotional and behavioral responses, as well as a single item to capture impairment of sound sensitivities broadly rather than misophonia specifically. Since the current study began, several new measures of misophonia have been validated that have more comprehensive assessment and stronger psychometric support [e.g., S-5 (Vitoratou et al., 2021); Misoquest (Siepsiak et al., 2020); Duke Misophonia Questionnaire (Rosenthal et al., 2021); Duke-Vanderbilt Misophonia Screening Questionnaire (Williams et al., 2022)]. Although we used the Duke Misophonia Questionnaire (Rosenthal et al., 2021) in this study, we did not administer it on a weekly basis to reduce patient burden and therefore could not include it in our outcomes measures analyses. Future work on changes in misophonia before, during, and after treatments should include additional measure of misophonia that are both sensitive to change and have strong face validity. Because of these limitations in measurement, we are limited in our ability to draw inferential conclusions about how misophonia as a comprehensive construct beyond its measurement in the MQ changed across treatment. Future work should include measures that have evidenced strong discriminant validity for sensory intolerance conditions that often co-occur with misophonia (e.g., hyperacusis, sensory over-responsivity). Lastly, the OANSIS is also not an empirically validated measure of anger. This measure was used in this study because instead of focusing on externalizing symptoms often assessed by other anger measures (e.g., yelling), it focused more on functional impairment (e.g., how often did you avoid situations, places, objects or activity because of anger?).

Despite these limitations, this is the first study to our knowledge that used this approach to demonstrate initial promise for using the UP as a treatment for misophonia in adults. Because it can be delivered by an individual therapist in 16 sessions in a wide range of clinical settings, this treatment provides an accessible and effective solution for patients struggling with misophonia. In line with previous findings (review of treatment studies), learning skills to manage emotional responses to misophonia can improve their symptoms and daily functioning. In this study, the UP helped patients learn to tolerate their discomfort in their misophonia contexts and use specific skills to react to the situation in heathier ways. Patients who were unable to join family dinners for years reported hardly even noticing their trigger sounds at the dinner table by the end of treatment. Although larger studies are warranted, this study offers hope that the UP is a viable treatment option for those suffering from misophonia.

## Data availability statement

The datasets presented in this article are not readily available because we will comply with Duke University Medical Center’s policies regarding sharing clinical research data. Requests to access the datasets should be directed to mark.rosenthal@duke.edu.

## Ethics statement

The studies involving humans were approved by DUHS Institutional Review Board. The studies were conducted in accordance with the local legislation and institutional requirements. The participants provided their written informed consent to participate in this study.

## Author contributions

KM: Conceptualization, Formal analysis, Investigation, Project administration, Writing – original draft, Writing – review & editing. CC-R: Conceptualization, Data curation, Funding acquisition, Investigation, Methodology, Project administration, Resources, Writing – review & editing. AG: Formal analysis, Methodology, Visualization, Writing – review & editing. RG: Formal analysis, Visualization, Writing – review & editing. EF-A: Visualization, Writing – review & editing, Data curation. LK: Data curation, Project administration, Writing – review & editing. MR: Conceptualization, Funding acquisition, Investigation, Methodology, Supervision, Writing – original draft.

## Footnotes

^1^ www.misophonia.duke.edu

^2^ www.unifiedprotocol.com
