# Supplementary material for: The unified protocol for transdiagnostic treatment of emotional disorders for misophonia: a pilot trial exploring acceptability and efficacy
Source: Front Psychol. 2024 Feb 9;14:1294571. doi: 10.3389/fpsyg.2023.1294571 (PMC10885161; doi:10.3389/fpsyg.2023.1294571)
Supplement: Supplementary file 1 [file Table_1.docx]

**Supplementary Tables**

*Themes from Qualitative Analysis of Acceptability Questionnaire for Study 1*

| **Question** | **Theme** | **Example Quote (from assessors notes or patient’s responses)** |
| --- | --- | --- |
| What they thought about the treatment overall | The treatment was worthwhile completing, helpful, and valuable as it resulted in many positive changes | Absolutely thinks it was worthwhile and does not regret doing it |
|  | They learned a lot about misophonia and how it affects their lives | Overall, it showed her that misophonia plays a larger role than she realized before treatment |
|  | The treatment taught useful skills that had impact on experiences beyond misophonia (e.g., anxiety) | Also realized the interrelatedness of the issues core to misophonia with other ongoing psychological targets. Learned things she can do to manage anxiety (i.e., slow down, walk away from the trigger) |
|  | They felt that their skills for managing misophonia had improved as a result of the treatment | Realized her coping mechanisms she relied on for her whole life may not be in best interest in long-term; realized she was dependent on headphones. Alternative actions; labelling emotional experiences. Learning not to rely on first automatic thought (b/c was always negative). |
|  | Some patients voiced dissatisfaction with elements of the treatment in response to this question, including not liking the basic nature of the skills or feeling frustrated with structured therapy that didn’t allow for venting | There were times that were frustrating because of the structured nature of therapy (i.e., not being able to vent for an hour) |
| Most helpful parts of treatment | The individual support from the therapist | [Therapist's name] very good at reducing her guilt and pointing out her improvement. Could tell [therapist’s name] really cared. |
|  | Homework exercises and worksheets that helped them track their emotions | Exercises during the week. As a visual person, the home practice made her write things down and analyze the context more logically rather than emotionally. Especially the ones that broke down each emotion. |
|  | Learning and practicing new skills | Learning what triggers were (esp. which were worse) and options for how to manage them (e.g., breathing, re-focusing, self-validation). |
|  | Meeting once a week | Weekly one-on-session were important for structure, to help keep her accountable. |
| Changes they would recommend | The option of receiving the treatment in-person or in a hybrid model instead of only telehealth, and that they could have the option to extend the treatment beyond 16 sessions to discuss other  personal issues or to further practice skills. | Possibly add a couple more weeks to the treatment, just in case an individual wants to take more time on a topic but doesn’t want to rush through the others. Even though there are 4 weeks available, if someone was like me, life would manage to get in the way each of those additional weeks. |
| Most important things they learned in treatment | It is possible to get better and reduce symptoms of misophonia | I learned that sounds are a major obstacle in my life but it can be better with work. |
|  | They can use the mindfulness,  cognitive, and behavioral skills to manage feelings in response to misophonia | I learned to control some of my feelings and how to refocus myself when a misphonia episode happens. |
|  | It’s important to accept emotions | It’s okay to feel angry, anxious, or upset. |

*Themes from Qualitative Analysis of Acceptability Questionnaire for Study 1*

| **Question** | **Theme** | **Example Quote (from assessors notes or patient’s responses)** |
| --- | --- | --- |
| What they thought about the treatment overall | Treatment was worthwhile, helpful and extremely valuable  as it resulted in many positive changes, many that were unexpected | This treatment has changed my life for the better in many ways |
|  | The treatment taught very  useful information and skills that had impact beyond misophonia (e.g., anxiety) | My emotional regulation and ability to process my anger, anxiety, and depression in a productive way have been enhanced. |
|  | They felt that their skills for managing misophonia had improved as a result of the treatment | My misophonia feels more manageable |
|  | Some patients had some complaints about the treatment, including not knowing how to balance  discussing other personal issues and misophonia symptoms during treatment | After a certain point I felt like I was only able to talk about my misophonia, I felt perhaps I spoke too much about other aspects of my life but in the beginning I was told that it was ok to do that. I know these studies are focusing on one diagnosis so it is challenging when other issues come up. |
| Most helpful parts of treatment | The individual support  of the therapist | My therapist [therapist’s name] was overall the most helpful part of the therapy. I don’t believe I could have gotten the same results reading a book on Unified Protocol. With the support, guidance, accountability, and empathy I received ; I was able to focus on making dramatic changes. |
|  | Homework exercises and worksheets | Throughout the treatment, the HW sheets were a good guide and reflection component |
|  | Learning and practicing new skills | Learning how to apply the various tools |
|  | Learning about emotions, how they work and how to identify them | Understanding how emotions work and re-defining my relationship to them as neutral was where I felt a shift |
|  | Learning about cognitive flexibility skills | Cognitive flexibility skills to help deal with misophonia |
|  | Practicing exposures | The exposure practices were huge for me |
|  | Meeting once a week | One on one therapy sessions to work through questions and practice |
| Specific changes in misophonia symptoms because of the treatment | Reduced levels of emotions in general and in response to triggers | On a normal day, I no longer respond to chewing with anxiety and anger. I have experienced mild disgust at times, but I am able to acknowledge it and continue on without a negative response. |
|  | Reduced emotional and physical misophonia symptoms | Physical symptoms (shortness of breath and muscle tension) were somewhat lessened. |
|  | More manageable symptoms as a  result of being less reactive to triggers and being able to generate alternative thoughts or actions | I had one mysophonia instance in the last month when I was feeling stressed out after taking the whole family to the pool. My daughter was chewing food loudly and I felt some anxiety. I was able to acknowledge my feelings before acting. I politely asked her if she would finish her snack. She did and after a few minutes I was feeling relaxed after a stressful day. |
| Changes they observed in their social interactions or relationships because of the treatment | Can spend time with triggering people and settings, including mealtimes | I am able to have dinner with people (which I was unable to do previously). It is still a struggle but a manageable struggle now. |
|  | That it was easier to engage in social interactions and relationships | Relationships are richer. I’m not isolating like I used to do. I actually go out for girls night. Part of this is better symptom coping. |
|  | That relationships at home have improved so that they can enjoy their family’s company (including pets and children) | My home life is so much better, too. I no longer live on the opposite side of my house behind a closed door wearing headphones. I am not letting the foot tapping, sniffling, etc., interfere with my ability to enjoy my family’s company. |
|  | They experienced more positive interactions with others because emotions are less intense | I feel less angry when a person creates a sound that is also a misophonic sound. I am therefore less irritable and my interactions are more positive. |
| Other changes in their lives or problems because of the treatment | Better understand what they’re feeling and why | My ability to break down what I am feeling and why has been enhanced by this treatment. |
|  | The ability to apply  skills to manage other symptoms like grief, depression, and anxiety | I was able to apply the CBT skills to other areas of life such as grief processing and anxiety |
|  | Increased hope for  the future | Also, because of treatment I was able to have new opportunities that have made me feel more secure and hopeful for the future. |
| Changes they would recommend | Option to add more time to spend on specific skills or other topics, particularly exposures | Longer time spent with emotions and feelings. That was the part that was the most challenging for me and also the section I got the most out of. |
|  | Not having any other major suggestions for ways to change the treatment | Nothing comes to mind! |
| Most important things they learned in treatment | It is possible to get better and have reduced misophonia symptoms | I learned that my misophonia can be managed! |
|  | They can use skills to manage misophonia | How to reapply cognitive flexibility skills to misophonia |
|  | The role and function of emotions | That emotions are an information and warning system. They are neutral and necessary. I ignored my emotions and constantly felt anxiety and anger. I am aware of my thoughts, feelings, and behaviors now. I understand the interactions and how to choose how to respond most of the time. Without this change, the other steps would not have been possible. |
|  | That they are capable of handling more than they think they could | I learned a lot about myself I am strong I am not powerless I have a voice I have choices I am not a victim I am capable. The world will not crumble and break away if I come across a sound or situation that’s uncomfortable |
